# Lutein, a Natural Carotenoid, Induces α-1,3-Glucan Accumulation on the Cell Wall Surface of Fungal Plant Pathogens

**DOI:** 10.3390/molecules21080980

**Published:** 2016-07-28

**Authors:** Junnosuke Otaka, Shigemi Seo, Marie Nishimura

**Affiliations:** Institute of Agrobiological Sciences, National Agriculture and Food Research Organization, 2-1-2 Kannondai, Tsukuba, Ibaraki 305-8602, Japan; o.junnosuke@gmail.com (J.O.); sseo71@affrc.go.jp (S.S.)

**Keywords:** α-1,3-glucan, bioassay-guided isolation, carotenoid, cell wall, fungal pathogen, sterol

## Abstract

α-1,3-Glucan, a component of the fungal cell wall, is a refractory polysaccharide for most plants. Previously, we showed that various fungal plant pathogens masked their cell wall surfaces with α-1,3-glucan to evade plant immunity. This surface accumulation of α-1,3-glucan was infection specific, suggesting that plant factors might induce its production in fungi. Through immunofluorescence observations of fungal cell walls, we found that carrot (*Daucus carota*) extract induced the accumulation of α-1,3-glucan on germlings in *Colletotrichum fioriniae*, a polyphagous fungal pathogen that causes anthracnose disease in various dicot plants. Bioassay-guided fractionation of carrot leaf extract successfully identified two active substances that caused α-1,3-glucan accumulation in this fungus: lutein, a carotenoid widely distributed in plants, and stigmasterol, a plant-specific membrane component. Lutein, which had a greater effect on *C. fioriniae*, also induced α-1,3-glucan accumulation in other *Colletotrichum* species and in the phylogenetically distant rice pathogen *Cochliobolus miyabeanus*, but not in the rice pathogen *Magnaporthe oryzae* belonging to the same phylogenetic subclass as *Colletotrichum*. Our results suggested that fungal plant pathogens reorganize their cell wall components in response to specific plant-derived compounds, which these pathogens may encounter during infection.

## 1. Introduction

Cell walls are essential for fungi to maintain their structure and to protect the cells from the environment. The fungal cell wall is mainly composed of polysaccharides such as glucans, chitin/chitosan and mannan. In filamentous ascomycete fungi, the structural core of the cell wall is generally composed of branched β-1,3-glucan and chitin. This core is embedded in amorphous polysaccharides, e.g., α-1,3-glucan, and the composition of the amorphous polysaccharides may vary by fungal class/genus [1,2,3]. Intensive studies on medically important fungi have revealed that the composition and localization of cell wall polysaccharides change in response to environmental conditions such as temperature [3]. Recently, we have performed immunohistochemical studies of the organization of cell wall polysaccharides in phylogenetically distant plant pathogenic fungi: the ascomycetes *Magnaporthe oryzae* and *Cochliobolus miyabeanus* and the basidiomycete *Rhizoctonia solani* [4,5]. Our studies revealed that these fungi accumulate α-1,3-glucan on their surfaces to mask the cell walls during infection. Because α-1,3-glucan is a refractory polysaccharide for plants, it is very likely that the surface accumulation of α-1,3-glucan protects the fungal cell walls from plant antifungal agents, such as cell wall digesting enzymes, which fungi encounter during infection [4]. This idea was supported experimentally; *M. oryzae* lacking surface α-1,3-glucan was very sensitive to a cell digesting enzyme. In addition, transgenic rice plants expressing a bacterial α-1,3-glucanase exhibited resistance towards *M. oryzae*, *C. miyabeanus*, and *R. solani* [5]. These results indicated that surface accumulation of α-1,3-glucan facilitates infection in these pathogenic fungi. We were interested in the fact that these fungal plant pathogens accumulate α-1,3-glucan on the cell wall specifically during infection but not during vegetative growth [4,5]. This implied that the fungi recognize host factor(s) to induce the surface accumulation of α-1,3-glucan. Indeed, we found that recognition of a cutin monomer, 1,16-hexadecanediol, induced the accumulation of α-1,3-glucan on the cell wall surface of *M. oryzae* by activating the cell wall integrity mitogen-activated protein kinase signal transduction pathway [4]. However, the cutin monomer caused very little surface accumulation of α-1,3-glucan in *C. miyabeanus* and *R. solani* [5], suggesting that these two pathogens respond to unknown plant factor(s). In this study, we describe the isolation and identification of novel host factors that induce the surface accumulation of α-1,3-glucan in the fungal plant pathogens such as *C. miyabeanus* and *Colletotrichum* species.

## 2. Results

### 2.1. Isolation and Identification of Inducers of α-1,3-Glucan Accumulation in Colletotrichum Fioriniae from Carrot Leaves Subsection

**Figure 1:**
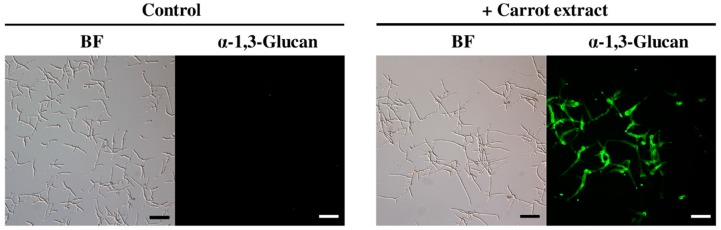
Immunofluorescent detection of α-1,3-glucan on the cell wall of *Colletotrichum fioriniae*. α-1,3-glucan was clearly visible on the cell wall of the fungi incubated in 0.24% potato dextrose broth (PDB) containing carrot extract (in 1% ethanol and 0.1% dimethyl sulfoxide (DMSO)). The 0.24% PDB solution containing 1% ethanol and 0.1% DMSO was used as a control. BF (**left panels**), bright field microscopy; “α-1,3-glucan” (**right panels**), fluorescence microscopy. Scale bar, 20 μm. More than 300 germinated fungal conidia were observed; representative images are shown. Experiments were repeated three times.

In the course of our immunohistological study of fungal cell walls, we noticed that *Colletotrichum fioriniae*, which causes anthracnose in a broad range of dicot plants [6,7], accumulated detectable amounts of α-1,3-glucan on the cell wall when the fungus was grown in medium containing carrot leaf extract (Figure 1). In contrast, α-1,3-glucan was hardly detected in the fungal cell wall when the fungus was incubated in medium that did not contain carrot extract (Figure 1).

**Figure 2:**
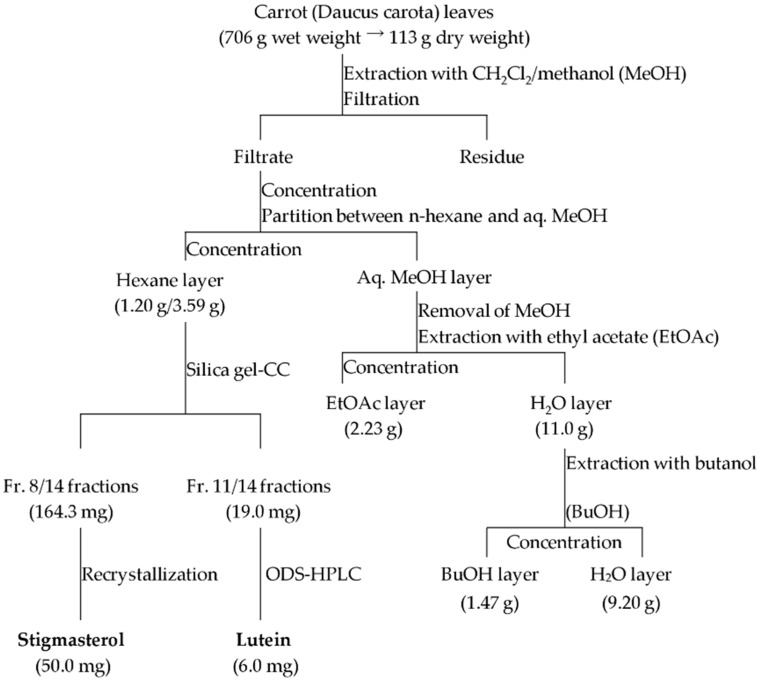
Isolation procedure of active substances from carrot leaves. Each fraction was tested for induction activity of α-1,3-glucan on the surface in *Colletotrichum fioriniae*.

We confirmed that 1,16-hexadecanediol, a known inducer of α-1,3-glucan accumulation in *M. oryzae* [4], did not cause the accumulation of α-1,3-glucan on the *C. fioriniae* cell wall (Figure S1). Based on these findings, we speculated that carrot leaf extract might contain novel inducer(s) of α-1,3-glucan accumulation that affect *C. fioriniae*. To identify the active substance(s) responsible for this α-1,3-glucan accumulation, we performed bioassay-guided fractionation of the carrot leaf extract. The lyophilized carrot leaves were extracted with a dichloromethane-methanol mixture and divided into hexane-, ethyl acetate-, butanol- and water-soluble layers (Figure 2).

**Figure 3:**
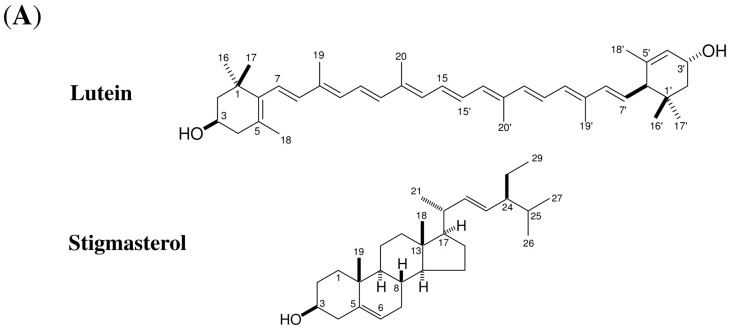

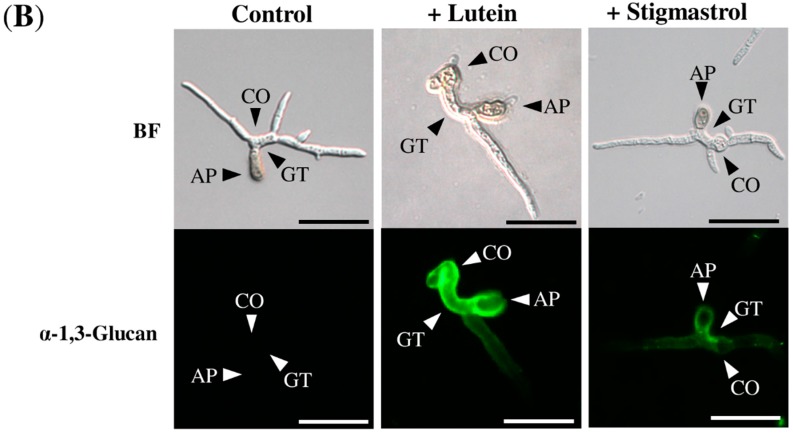
Immunofluorescent detection of α-1,3-glucan on the cell wall of *Colletotrichum fioriniae* in the presence of active substances from carrot leaves. (**A**) The structures of lutein and stigmasterol; (**B**) Accumulation of surface α-1,3-glucan in the presence of lutein (10 μM) and stigmasterol (50 μM). The fungal conidia were incubated in 0.24% potato dextrose broth (PDB) containing lutein/stigmasterol dissolved in 1% ethanol and 0.1% dimethyl sulfoxide (DMSO). Control; 0.24% PDB solution containing 1% ethanol and 0.1% DMSO. BF, bright field microscopy; “α-1,3-glucan”, fluorescence microscopy. AP, appressoria; CO, conidia; GT, germ tubes. Scale bar, 20 μm. More than 300 germinated fungal conidia were observed for each sample. Representative images at 12 h after incubation are shown. Experiments were repeated three times.

Each layer was mixed with fungal conidia and incubated on a cover glass, and the accumulation of α-1,3-glucan on the fungal cell wall was observed using a fluorophore-conjugated α-1,3-glucan antibody. The strongest activity was found in the hexane layer; this activity was separated into two active fractions using silica gel chromatography. Further purification of the two fractions led to the isolation of an active compound for each. ^1^H- and ^13^C-NMR analyses revealed that the active compound with the highest activity was lutein (Figure 3; Figure S4), and the modest activity was assigned to stigmasterol (Figure 3).

We further tested whether β-carotene and ergosterol, structurally similar compounds to lutein and stigmasterol, respectively, induced surface α-1,3-glucan accumulation in *C. fioriniae*. As shown in Figure S2, α-1,3-glucan accumulation was undetectable in the presence of β-carotene and low in the presence of ergosterol. This suggested that *C. fioriniae* specifically responded to lutein.

### 2.2. Lutein Induced the Accumulation of α-1,3-Glucan on the Surface in Various Colletotrichum Species

**Figure 4:**
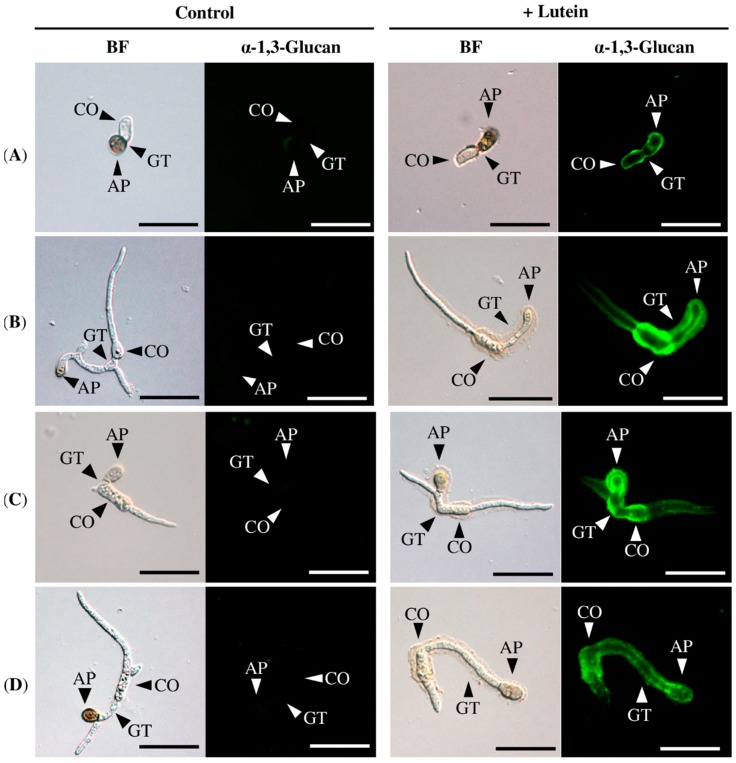
Surface accumulation of α-1,3-glucan on the cell walls of *Colletotrichum* species in the presence of lutein. (**A**) *C. orbiculare*, (**B**) *C. gloeosporoides*, (**C**) *C. chrysanthemi*, (**D**) *C. godetiae*. Fungal conidia were incubated in 0.24% potato dextrose broth (PDB) containing lutein (25 μM in 1% ethanol and 0.1% dimethyl sulfoxide [DMSO]) for 12 h and subjected to immunofluorescent detection of cell wall α-1,3-glucan. Control, 0.24% PDB containing 1% ethanol and 0.1% DMSO. BF, bright field microscopy; ‘’α-1,3-glucan‘’, fluorescence microscopy. AP, appressoria; CO, conidia; GT, germ tube. Scale bar, 20 μm. More than 300 germinated fungal conidia were observed for each sample. Representative images at 12 h after incubation are shown. Experiments were repeated three times.

To examine the inductive effect of lutein in other *Colletotrichum* species, we incubated conidia of *C. orbiculare*, *C. gloeosporioides*, *C. chrysanthemi*, and *C. godetiae* with or without lutein on glass coverslips, and appressorium-forming conidia were immunofluorescently stained for α-1,3-glucan on the cell wall. As shown in Figure 4, fluorescence was hardly detected on the cell walls of appressorium-forming conidia in these *Colletotrichum* strains when they were incubated in the absence of lutein. In contrast, fluorescence was detectable on those incubated with lutein, although the fluorescence was most intense on *C. gloeosporioides*, *C. chrysanthemi* and *C. godetiae*, and relatively weak on *C. orbiculare* (Figure 4).

Similar to *C. fioriniae*, the species *C. gloeosporioides* and *C. godetiae* infect a broad range of plants. *C. chrysanthemi and C. orbiculare*, in contrast, show preferences for asterids and cucurbits, respectively [6,7,8]. This result indicated that the surface accumulation of α-1,3-glucan was induced by lutein in these *Colletotrichum* species regardless of the preferred host plants. 

### 2.3. Lutein Induced α-1,3-Glucan Accumulation in Cochliobolus Miyabeanus but Not in Magnaporthe oryzae

**Figure 5:**
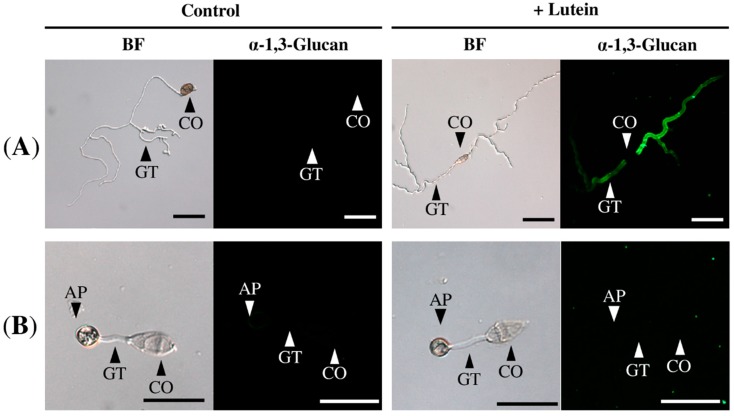
Effect of lutein on the accumulation of α-1,3-glucan on the cell walls of *Cochliobolus miyabeanus* and *Magnaporthe oryzae*. (**A**) *C. miyabeanus*; (**B**) *M. oryzae*. Fungal conidia were incubated in 0.24% potato dextrose broth (PDB) containing stigmasterol (25 μM in 1% ethanol and 0.1% dimethyl sulfoxide [DMSO]). Control, 0.24% PDB containing 1% ethanol and 0.1% DMSO. *C. miyabeanus* accumulated α-1,3-glucan on the cell wall in response to lutein, whereas *M. oryzae* did not. BF, bright field microscopy; “α-1,3-glucan‘’, fluorescence microscopy. AP, appressoria; CO, conidia; GT, germ tubes. Scale bar, 20 μm. More than 300 germinated fungal conidia were observed for each sample. Representative images at 12 h after incubation are shown. Experiments were repeated three times.

Because lutein is the most abundant carotenoid in photosynthetic plant tissues [9], we further tested whether lutein induced α-1,3-glucan accumulation on *M. oryzae* and *C. miyabeanus*, both of which accumulated α-1,3-glucan during rice infection [4,5]. Because *M. oryzae* belongs to same subclass as *Colletotrichum* but *C. miyabeanus* is phylogenetically distant from *Colletotrichum*, we speculated that *M. oryzae* could respond to lutein but not *C. miyabeanus*. When *M. oryzae* and *C. miyabeanus* conidia were incubated on glass coverslips with or without lutein, unexpectedly, cell wall α-1,3-glucan was undetectable in *M. oryzae* but was present in large amounts in *C. miyabeanus* based on the immunofluorescence staining (Figure 5). Thus, *C. miyabeanus*, but not *M. oryzae*, responded to lutein as well as Colletotrichum strains to accumulate α-1,3-glucan on their surface.

### 2.4. Stigmasterol Showed Induction Activity on Surface α-1,3-Glucan Accumulation in Various Fungi

Stigmasterol is a major component of plant membranes [10] that fungal plant pathogens may encounter during infection. To examine whether the induction effect was specific to *C. fioriniae*, α-1,3-glucan on the cell wall surfaces of *M. oryzae* and *C. miyabeanus* incubated in the presence of stigmasterol was detected. Similar to the result obtained with *C. fioriniae*, stigmasterol induced the surface accumulation of α-1,3-glucan in these fungi (Figure S3). These results suggested that stigmasterol is a common inducer of surface α-1,3-glucan accumulation in various fungal plant pathogens.

### 2.5. Surface Accumulation of α-1,3-Glucan Protected the Cell Wall from Digestive Enzymes in Colletotrichum Fioriniae

**Figure 6:**
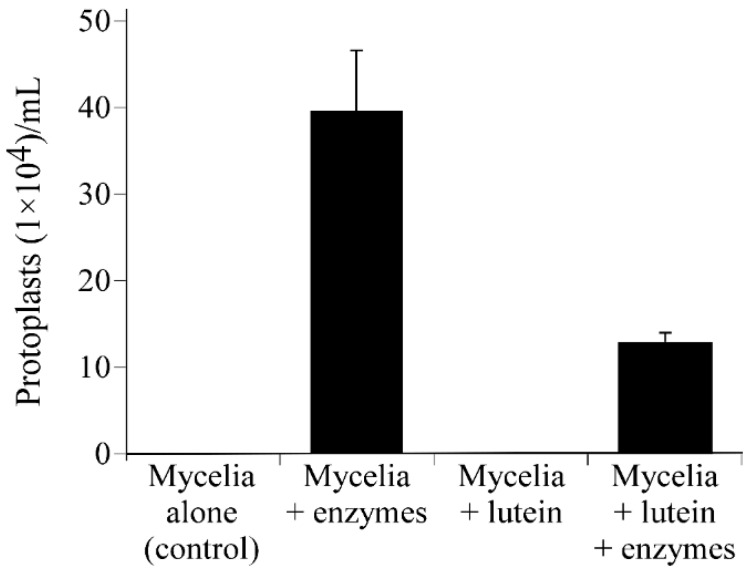
Sensitivity assay to a cell-wall-digesting enzyme cocktail. Number of protoplasts released from *Colletotrichum fioriniae* cell walls after enzymatic digestion using Uskizyme (a cocktail of β-1,3-glucanase and chitinase). + enzyme, mycelial samples treated with Uskizyme (0.5 mg/mL) for 24 h; + lutein, fungal mycelia incubated with lutein (20 μM) for 24 h prior to the enzymatic digestion. The fungal mycelia incubated with lutein released lower numbers of protoplasts after enzymatic treatment.

The surface accumulated α-1,3-glucan protected the cell wall from digestive enzymes in *M. oryzae* [5]. To test whether the surface α-1,3-glucan protected the cell wall in *C. fioriniae*, we treated the fungal mycelia with Uskizyme (Wako Chemicals, Tokyo, Japan), a cocktail of chitinase and β-1,3-glucanase. As shown in Figure 6, the mycelia grown in the presence of lutein released fewer protoplasts after this enzymatic treatment than those grown in the absence of lutein. This result suggested that the surface accumulation of α-1,3-glucan protected the fungal cell wall from digestive enzymes.

## 3. Discussion

Fungi are known to reorganize their cell wall components in response to cell wall and plasma membrane perturbation [3,11,12]. In this study we identified lutein and stigmasterol, compounds broadly present in plants, as inducers of cell wall reorganization in *C. fioriniae*; lutein showed greater induction activity than stigmasterol. Notably, lutein induced cell wall reorganization in various *Colletotrichum* species and *C. miyabeanus* but hardly in *M. oryzae*. The fact that the response to lutein was different in these fungi suggesting that presence of lutein is not a general stress factor for the fungal cell wall and the plasma membrane. Similarly, responses to the plant cutin monomer 1,16-hexadecanediol varied in different fungi [4,5]; reorganization of cell wall components was clearly observed with *M. oryzae* [4] but hardly with *C. miyabeanus* [5] and *C. fioriniae*. Of note, β-carotene was not as effective as lutein in *C. fioriniae* in terms of inducing cell wall reorganization, although these compounds are structurally similar to each other. This result indicated that the difference in the chemical structure between lutein and β-carotene affected the fungal response. These results would indicate that fungal plant pathogens have mechanisms that allow the fungi to sense specific compounds released from plants.

In contrast to lutein, stigmasterol induced the surface accumulation of α-1,3-glucan in various fungi including *M. oryzae*. In addition, ergosterol, a close analog of stigmasterol, showed similar activity to stigmasterol in *C. fioriniae*. These results suggested that presence of exogenous stigmasterol and its structurally related compounds act as general inducers of cell wall reorganization in fungi. Perturbation of the plasma membrane caused by insertion of amphipathic molecules into the membrane activates cell wall integrity signaling, which regulate the synthesis and reorganization of the cell wall in yeasts [11]. Stigmasterol and ergosterol are amphipathic components of plasma membranes in plant and fungi, respectively [10]. Together, these amphipathic sterols could cause perturbation in the plasma membrane, thereby activating cell wall integrity signaling in these fungal plant pathogens.

Given that these pathogens specifically recognize lutein during infection, how do they access the lutein synthesized and accumulated in plastids in plant cells? It is notable that the infectious life styles of *Colletotrichum* species are hemibiotrophic, and *C. miyabeanus* is necrotrophic; these pathogens kill host cells at a certain point during infection [8,13]. Lutein could be released from plastids that collapsed as a consequence of fungal killing of the host plant cells. Considering that antifungal enzymes and compounds leak from collapsed vacuoles in dead plant cells [12], the surface α-1,3-glucan could facilitate fungal survival by protecting fungal cells during necrotrophic colonization of host cells. Indeed, sensitivity assays against the cell wall digestive enzymes performed in this study indicated that surface α-1,3-glucan accumulation protected the fungal cell wall from chitinase and β-1,3-glucanse, the major antifungal enzymes in plants [14]. Stigmasterol released from the plant membrane upon fungal penetration or the membranous collapse of dead plant cells could also facilitate fungal survival during infection via promoting the accumulation of surface α-1,3-glucan. Overall, our study suggests that fungal plant pathogens respond to specific factors in plants and modulate their cell wall organization upon infection.

## 4. Experimental Section

### 4.1. Fungal Strains and Plant Materials

All *Colletotrichum* strains (*C. chrysanthemi* (MAFF 239363), *C. orbiculare* (MAFF 240422), *C. godetiae* (MAFF 241297), *C. gloeosporioides* (MAFF 306533), *C. fioriniae* (MAFF 306551)) and *C. miyabeanus* (MAFF 305425) were obtained from the MAFF gene bank in National Institute of Agrobiological Sciences (Tsukuba, Ibaraki, Japan). Wild-type *M. oryzae* Guy11 was used for this study. Commercial fresh carrots were supplied as materials for this study.

### 4.2. Chemicals

Lutein and 1,16-hexadecanediol were purchased from United States Biological (Salem, MA, USA) and Tokyo Chemical Industry (Tokyo, Japan), respectively.

### 4.3. General

^1^H- and ^13^C-NMR (500 MHz, 125 MHz) spectra were recorded on an AV-500 NMR spectrometer (Bruker, Billerica, MA, USA) with CDCl_3_ as the solvent. The chemical shifts are reported in ppm (parts per million; *δ*), and coupling constants (*J*) are expressed in Hz. Thin layer chromatography (TLC) was carried out on silica gel 60 F254 (250 μm, Merck, Darmstadt, Germany) or silica gel 60 RP-18 F254_S_ (250 μm, Merck). Flash column chromatography was carried out on silica gel 60 N (40–100 μm, Kanto Chemical, Tokyo, Japan). Reversed-phase high-performance liquid chromatography (RP-HPLC) was performed using a Waters 600 HPLC pump/2489 UV/VIS detector (Waters, Milford, MA, USA) computerized data station equipped with Waters Empower-2 software with a reversed-phase column CAPCELL PAK C_18_ (Shiseido, Tokyo, Japan). Microscopic observation was performed using a Leica DR System with a GFP filter cube (excitation filter BP 470/40 nm, 500 nm dichromatic mirror, suppression filter BP 525/50 nm) (Leica, Wetzlar, Germany).

### 4.4. Extraction and Isolation

Carrot leaves (706 g) were minced, lyophilized, and then extracted by immersing in CH_2_Cl_2_/methanol (MeOH) (1:1, 3 L) for 7 days at room temperature. The extract was filtered and concentrated under reduced pressure (“carrot extract”). The concentrated filtrate was partitioned twice between *n*-hexane (1 L) and 30% MeOH aqueous solution (1 L). The hexane layer was concentrated to obtain 3.59 g of syrup. The 30% MeOH layer was concentrated to remove MeOH, and the remaining aqueous suspension was partitioned twice with ethyl acetate (1 L). The ethyl acetate layer was evaporated under reduced pressure to obtain 2.23 g syrup. The aqueous residue was further partitioned with butanol (1 L) twice to give a BuOH extract (1.47 g) and H_2_O residue (9.20 g). A portion of the hexane layer (LH, 1.20 g/3.59 g) was subjected to silica gel flash column chromatography (50.0 g, 250 mm × 30 mm i.d., hexane–ethyl acetate, 20:1 (300 mL), 10:1 (300 mL), 5:1 (300 mL), 2:1 (400 mL), 1:1 (400 mL), and CH_2_Cl_2_–MeOH, 10:1 (200 mL), 2:1 (300 mL)), and fractionated into 20 mL each to obtain 100 fractions. On the basis of the TLC analyses, all fractions were collected to give 14 fractions (LH-1–14). Fraction LH-11 (19.0 mg), of which *R*_f_ value range was from 0.6 to 0.8 by CH_2_Cl_2_–MeOH 8:1, was further purified by RP-HPLC (CAPCELL PAK C18, 250 mm × 4.6 mm i.d., Shiseido, 97.5% acetonitrile), affording lutein (6.0 mg; *t*_R_: 14.3 min). Stigmasterol (50.0 mg) was isolated from fraction LH-8 (*R*_f_ value range was from 0.5 to 0.7 by hexane–ethyl acetate 2:1) via recrystallization twice using hexane and acetone.

*Lutein*: Orange powder; ^1^H-NMR (CDCl_3_) δ 6.66–6.57 (4H, m, H-11, 11’, 15, 15’), 6.35 (each 1H, d, *J* = 15.0 Hz, H-12, 12’), 6.25 (2H, m, H-14, 14’), 6.13–6.09 (5H, m, H-7, 8, 8’, 10, 10’), 5.54 (1H, brs, H-4’), 5.43 (1H, dd, *J* = 15.5, 9.5 Hz, H-7’), 4.23 (1H, m, H-3’), 4.00 (1H, m, H-3), 2.40 (1H, d, *J* = 9.5 Hz, H-6’), 2.35–2.28 (1H, m, H-4*eq*), 2.03 (1H, dd, *J* = 16.7, 9.7 Hz, H-4*ax*), 1.97 (3H, s, H-19), 1.96 (6H, brs, H-20, 20’), 1.91 (3H, s, H-19’), 1.83 (1H, dd, *J* = 13.5, 5.9 Hz, H-2’*eq*), 1.74 (1H, ddd, *J* = 12.0, 3.3, 2.1 Hz, H-2b), 1.73 (3H, s, H-18), 1.62 (3H, s, H-18’), 1.47 (1H, t, *J* = 12.0 Hz, H-2a), 1.36 (1H, dd, *J* = 13.5, 7.0 Hz, H-2’*ax*), 1.07 (6H, brs, C-16, 17), 0.99 (3H, s, C-16’), 0.85 (3H, s, H-17’); ^13^C-NMR (CDCl_3_) δ 138.6 (C-8), 137.9 (C-8’), 137.7 (C-5’), 137.6 (C-6, 9’, 12, 12’, 13), 136.5 (C-13’), 135.7 (C-9), 132.6 (C-14, 14’), 131.3 (C-10), 130.8 (C-10’), 130.0 (C-15, 15’), 128.7 (C-7’), 126.2 (C-5), 125.6 (C-7), 124.9 (C-4’), 124.9 (C-11), 124.5 (C-11’), 65.9 (C-3’), 65.1 (C-3), 55.0 (C-6’), 48.5 (C-2), 44.7 (C-2’), 42.6 (C-4), 37.1 (C-1), 34.0 (C-1’), 30.3 (C-17), 29.5 (C-16’), 28.7 (C-16), 24.3 (C-17’), 22.9 (C-18’), 21.6 (C-18), 13.1 (C-19’), 12.8 (C-19, 20, 20’) (Figure S4).

*Stigmasterol*: White powder; ^1^H-NMR (CDCl_3_) *δ* 5.34 (1H, brs, H-6), 5.14 (1H, dd, *J* = 15.0, 8.5 Hz, H-23), 5.01 (1H, dd, *J* = 15.0, 8.5 Hz, H-22), 3.52 (1H, tt, *J* = 11.0, 4.5 Hz, H-3), 2.30–2.21 (4H, m, H-4, 20, 24), 2.01–1.94 (3H, m, H-7, 25), 1.01–1.03 (6H, brs, H-19, 21), 0.92 (3H, d, 5.0 Hz, H-26), 0.83 (3H, dd, *J* = 7.5, 7.5 Hz, H-29), 0.81 (3H, d, *J* = 5.0 Hz, H-27), 0.67 (3H, s, H-18);^13^C-NMR (CDCl_3_) *δ* 140.8 (C-5), 138.3 (C-22), 129.3 (C-23), 121.7 (C-6), 71.8 (C-3), 56.8 (C-14), 56.1 (C-17), 51.2 (C-24), 50.2 (C-9), 45.6 (C-4), 42.3 (C-13), 40.5 (C-20), 39.8 (C-12), 37.3 (C-1), 36.5 (C-10), 36.2 (C-8), 33.9 (C-25), 32.0 (C-2, 7), 28.3 (C-16), 26.1 (C-28), 24.3 (C-15), 23.1 (C-21), 21.1 (C-26), 19.8 (C-14), 19.4 (C-19), 19.0 (C-27), 18.8 (C-11), 12.3 (C-29), 12.0 (C-18) (Figure S5).

### 4.5. Immunofluorescent Staining of Fungal Cell Wall α-1,3-Glucan

The *Colletotrichum* strains were grown on potato dextrose agar (PDA; BD Difco, Franklin Lakes, NJ, USA) at 25 °C with constant near-ultra violet (UV) irradiation for 5 days to induce conidiation. The *C. miyabeanus* strain was first grown on PDA at 25 °C with constant fluorescence for 5 days followed by further incubation at 20 °C with constant near-UV irradiation for 7 days to induce conidiation. The *M. oryzae* strain was incubated on oatmeal agar media (3% oatmeal and 1.6% agar) at 25 °C with constant fluorescence for 7 days to induce conidiation. The fungal conidia were suspended in 0.24% potato dextrose broth (PDB; BD Difco) aqueous solution at a concentration of 2 × 10^5^ conidia/mL. For the bioassays, aliquots of the samples from each fraction were dissolved in ethanol containing 10% dimethyl sulfoxide (DMSO) at a concentration of 1000 ppm. The sample solutions were added to conidial suspensions at a final concentration of 10 ppm (100-fold dilution). The sample/conidia mixture (50 μL) was dropped onto a cover glass (Matsunami, Osaka, Japan) and incubated for 12 h at 20 °C in a moist chamber in the dark. Fungal cells were then rinsed lightly with PBS buffer composed of NaCl (80.0 g/L), Na_2_HPO_4_∙12H_2_O (29.0 g/L), KCl (2.0 g/L), and KH_2_PO_4_ (2.0 g/L).

In the following assays, commercially available lutein and purified stigmasterol were used. To detect α-1,3-glucan on *Colletotrichum* strains, 30 μL of the primary antibody solution (0.1 mg/mL of IgMγ MOPC-104E (Sigma, St. Louis, MO, USA) in PBS buffer) was first dropped onto the fungal cells and incubated for 4 h at room temperature in the dark. The samples were rinsed with PBS buffer followed by a 1 h incubation with 20 μL fluorescein isothiocyanate (FITC)-conjugated secondary antibody (0.1 mg/mL of rabbit anti-mouse IgM, μ chain specific, Jackson ImmunoResearch, West Grove, PA, USA) in PBS buffer in the dark at room temperature. Detection of α-1,3-glucan on *M. oryzae* and *C. miyabeanus* was performed as described in Fujikawa et al. [4,5]. Samples were rinsed with PBS buffer before microscopic observation using a Leica DR system.

### 4.6. Sensitivity Assay to Cell Wall Digesting Enzymes

*C. fioriniae* conidia were incubated in PD medium (1 × 10^5^/mL) for 24 h followed by additional incubation for 24 h in the presence or absence of 20 μM lutein at 25 °C in the dark. The fungal mycelia were washed twice with 1.2 M sorbitol in 1 mM PIPES (pH 6.5) before being digested with Uskizyme (Wako, Osaka, Japan) (0.5 mg/mL 1.2 M sorbitol in 1 mM PIPES, pH 6.5) at 28 °C for 24 h in the dark. Uskizyme is an enzyme cocktail of >5000 units/g of β-1,3-glucanase and >30 units/g of chitinase. Numbers of generated protoplasts were counted using a hemocytometer.

## 5. Conclusions

Fungal plant pathogens reorganize their cell wall components in response to specific plant-derived compounds such as carotenoids.

## Supplementary Materials

Supplementary materials can be accessed at: http://www.mdpi.com/1420-3049/21/8/980/s1.
