# Supplementary material for: Lutein, a Natural Carotenoid, Induces α-1,3-Glucan Accumulation on the Cell Wall Surface of Fungal Plant Pathogens
Source: Molecules. 2016 Jul 28;21(8):980. doi: 10.3390/molecules21080980 (PMC6273161; doi:10.3390/molecules21080980)
Supplement: Supplementary file 1 [file molecules-21-00980-s001.pdf]

Supplementary Materials: Lutein, a Natural Carotenoid, Induces α-1,3-glucan Accumulation on the Surface of the Cell Wall in Fungal Plant Pathogens

Junnosuke Otaka, Shigemi Seo and Marie Nishimura

(**A**)

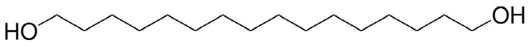

(**B**)

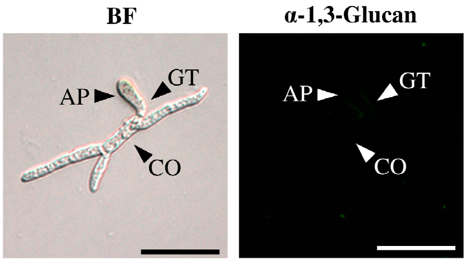

Figure S1. Effect of 1,16-hexadecanediol on the *Colletotrichum fioriniae* cell wall (**A**). The structure of 1,16-hexadecanediol. (**B**) Detection of α-1,3-glucan on the cell wall of *C. fioriniae* incubated in the presence of 1,16-hexadecanediol (50 μM in 1% ethanol and 0.1% dimethyl sulfoxide [DMSO]) in 0.24% potato dextrose broth (PDB) for 12 h. Control, 0.24% PDB containing 1% ethanol and 0.1% DMSO. No obvious α-1,3-glucan accumulation occurred on the fungal cell walls in response to 1,16-hexadecanediol. BF, bright field microscopy; 'α-1,3-glucan', fluorescence microscopy. AP, appressorium; CO, conidia; GT, germ tube. Scale bar, 20 μm. More than 300 germinated fungal conidia were observed for each sample. Representative images are shown. Experiments were repeated three times.

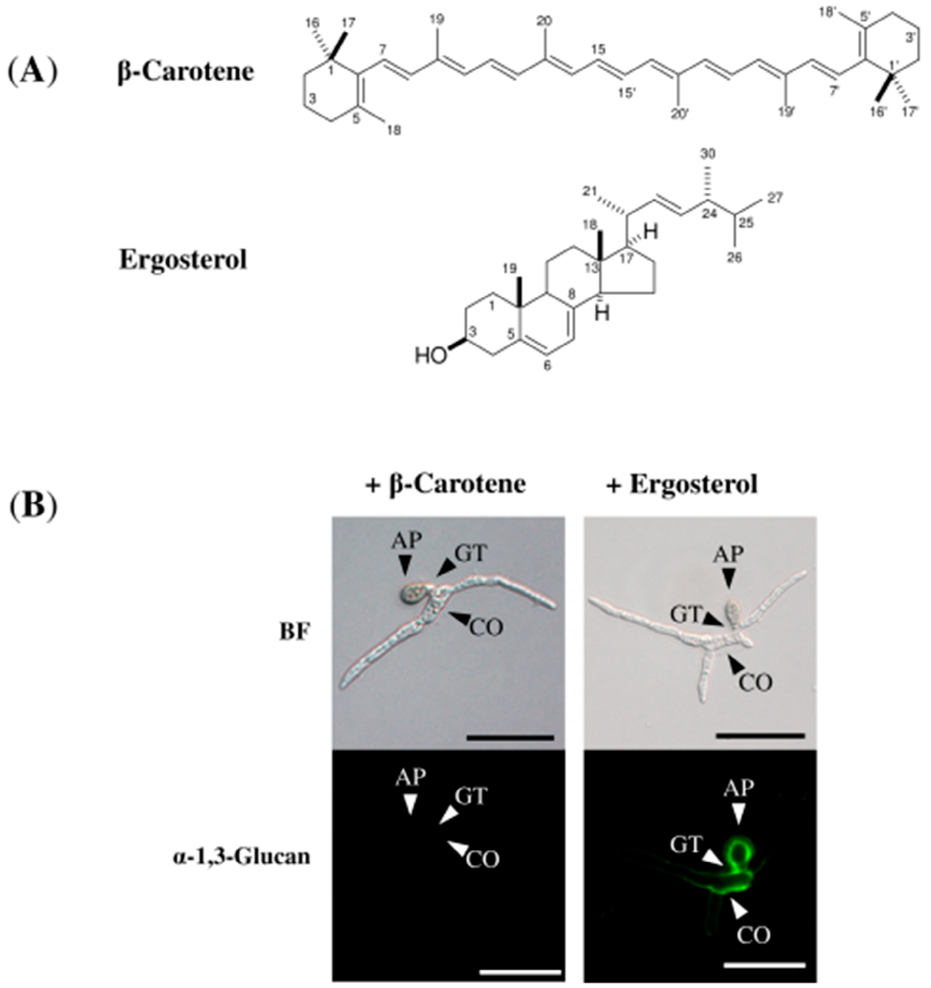

Figure S2. Immunofluorescent detection of α-1,3-glucan on the cell wall of *Colletotrichum fioriniae* in the presence of β-carotene and ergosterol. (**A**). The structures of β-carotene and ergosterol; (**B**) Detection of α-1,3-glucan on the cell wall of *C. fioriniae* incubated with β-carotene and ergosterol. *C. fioriniae* conidia were incubated for 12 h on cover glasses in 0.24% potato dextrose broth (PDB). For the control image, see Figure 3B. No obvious α-1,3-glucan accumulation was detected in the presence of β-carotene (100 μM in 1% ethanol and 0.1% dimethyl sulfoxide [DMSO]). In contrast, accumulation of α-1,3-glucan was observed, although weakly, in the presence of ergosterol (50 μM in 1% ethanol and 0.1% DMSO). BF, bright field microscopy; 'α-1,3-glucan', fluorescence microscopy. AP, appressorium; CO, conidia; GT, germ tube. Scale bar, 20 μm. More than 300 germinated fungal conidia were observed for each sample. Representative images are shown. Experiments were repeated three times.

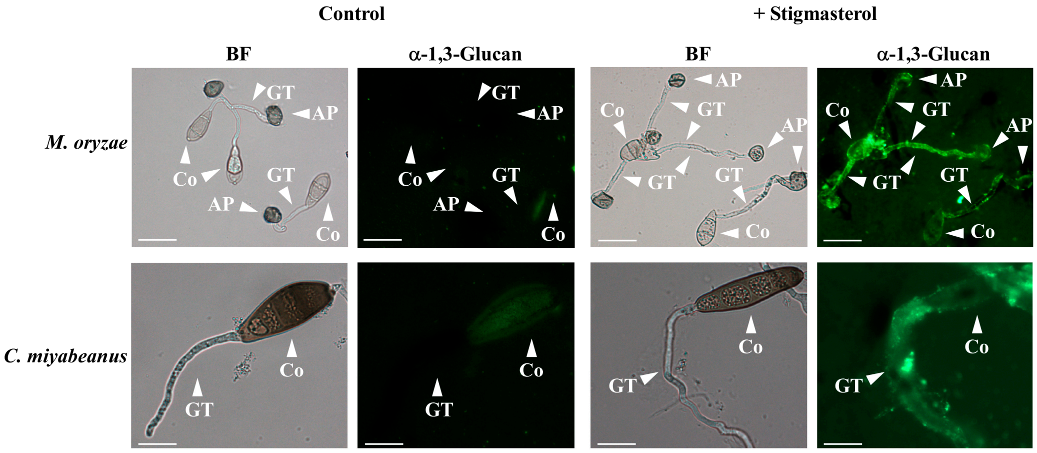

Figure S3. Effect of stigmasterol on accumulation of α-1,3-glucan on the cell walls of *Magnaporthe oryzae* and Cochliobolus miyabeanus. (**A**) *M. oryzae*, (**B**) *C. miyabeanus*. Fungal conidia were incubated in 0.24% potato dextrose broth (PDB) containing stigmasterol (50 μM in 1% ethanol and 0.1% dimethyl sulfoxide [DMSO]). Control, 0.24% PDB containing 1% ethanol and 0.1% DMSO. Both *C. miyabeanus* and *M. oryzae* accumulated α-1,3-glucan on the cell wall in the presence of stigmasterol. BF, bright field microscopy; 'α-1,3-glucan', fluorescence microscopy. AP, appressorium; CO, conidia; GT, germ tubes. Scale bar, 20 μm. More than 100 germinated fungal conidia were observed for each sample. Representative images at 12 h after incubation (hai) are shown. Experiments were repeated more than three times.

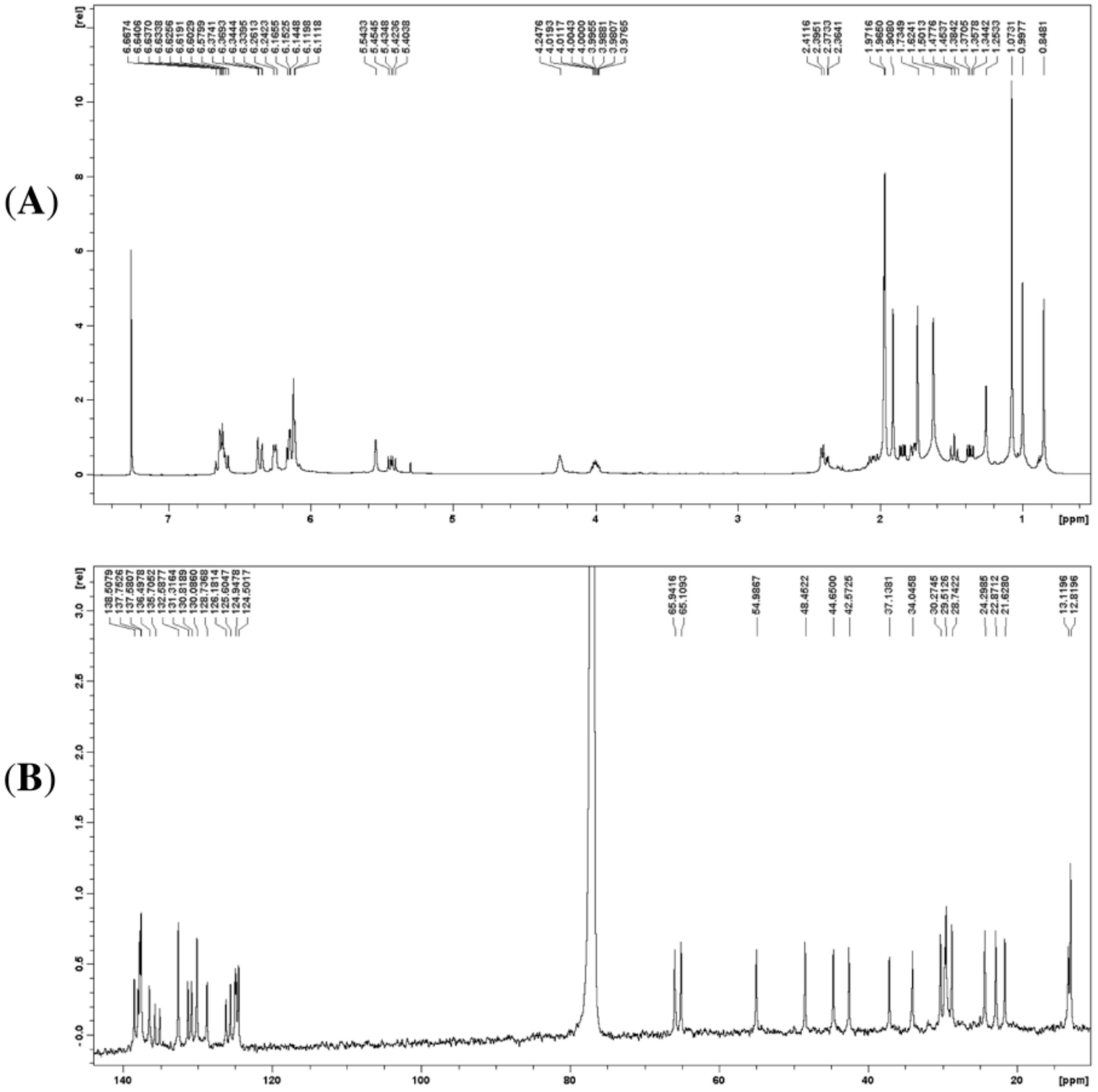

Figure S4. Nuclear magnetic resonance (NMR) spectra of lutein. (**A**) ^{1}H-NMR (500 MHz, in $CDCl_3$) spectrum of lutein isolated from fraction LH-11; (**B**) ^{13}C-NMR (125 MHz, in $CDCl_3$) spectrum of lutein isolated from fraction LH-11.

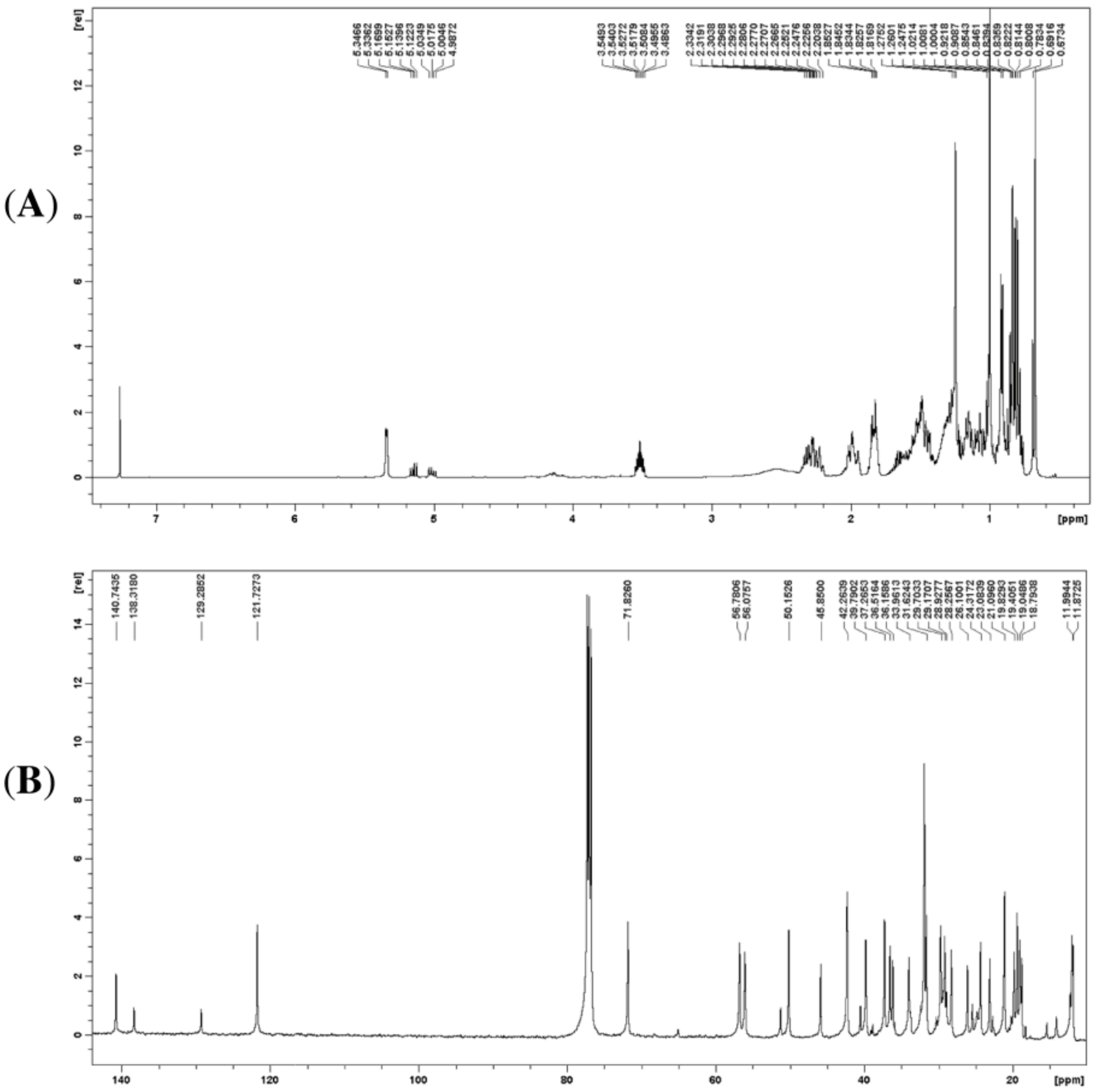

Figure S5. NMR spectra of stigmasterol. (**A**) ^{1}H-NMR (500 MHz, in $CDCl_3$) spectrum of stigmasterol isolated from fraction LH-8; (**B**) ^{13}C-NMR (125 MHz, in $CDCl_3$) spectrum of stigmasterol isolated from fraction LH-8.
